# Orthopedic trauma surgeon in Sierra Leone: how to keep one’s head over water

**DOI:** 10.1007/s00402-019-03171-1

**Published:** 2019-03-25

**Authors:** Florian Wichlas, Serafim Tsitsilonis, Michela Delli Guanti, Gino Strada, Christian Deininger

**Affiliations:** 1 Emergency NGO, Milan, Italy; 2 Center for Musculoskeletal Surgery, Charité-Universitätsmedizin Berlin, Augustenburger Platz 1, 13353 Berlin, Germany; 3 Universitätsklinik für Orthopädie und Traumatologie, Müllner Hauptstraße 48, Salzburg, 5020 Austria

**Keywords:** Trauma surgery, Low-income country, Africa, Third world, Guideline

## Abstract

**Purpose:**

Developing a guideline for orthopedic trauma surgeons working in civilian trauma hospitals in low-income countries.

**Methods:**

This is a retrospective data analysis in a non-governmental organizational trauma hospital in Sierra Leone, Africa. Trauma victims (282), with 349 fractures, were admitted to the hospital 10/2015–01/2016. The incidence of open and closed fractures and the use of implants were evaluated.

**Results:**

The most common fractures were open and closed tibial shaft fractures and closed femoral shaft fractures in adults, and closed supracondylar humerus fractures in children. The most used implants were external fixators, K-wires, and intramedullary nails. External fixators were used for open fractures, K-wires for closed fractures in children, hand and foot, and nails for closed fractures of the lower extremity in adults. Plates were used the least and mostly for fractures of the upper extremity, the proximal tibia and malleolar region in adults. The complication rate was 5.67%.

**Conclusion:**

Surgeons in low-income country trauma hospitals should treat conservatively on outpatient basis only, to reduce the amount of stationary patients. Open fractures should be treated with external fixators, and closed fractures in children, hand and foot, with K-wires. Closed fractures in adults of the lower extremity should be nailed, and closed fractures in adults of the upper extremity can be treated with plates.

## Introduction

Low-income countries (LIC) face a large increase of trauma patients resulting from civilian trauma caused by road traffic accidents [3, 9, 17]. Hospital capacities in LIC, on the other hand, are limited in terms of resources, infrastructure, and medical knowledge [6]. There is a low amount of hospitals and personnel that are not trained properly for the treatment of severely injured patients [4]. Non-governmental organizational (NGO) hospitals offer treatment for these patients free of charge. The latter fact may lead to even higher patient loads in these hospitals because Sierra Leone ranks 181 of the 188 nations on the United Nations Development Index [1]. International surgeons, working for these NGOs, treat patients, train the national staff and enable treatment protocols for patient care [18]. These protocols are needed to reach a comparatively even level of consistency, because the international experts change often. They face this high amount of severely injured patients in an uncommon environment and have to adapt to limited resources. The surgeons have to know common local injuries and how to treat them. Complicated and sophisticated treatment methods might not be indicated as patients’ compliance is low [13] and put treatment results at risk. Surgery should be simple, safe, and permit a high turnover. Solution strategies for this dilemma would be wishful and could guide international and unspecialized national surgeons.

We analyzed data of a single NGO trauma hospital for civilian trauma to identify factors that could be used to generate solution strategies for coping with the overwhelming amount of patients. Our goal was to propose an algorithm to guide surgeons in similar situations.

## Materials and methods

### Setting

The patients were acquired in an NGO hospital in Freetown, Sierra Leona, Africa. The hospital had 85 beds, eight intensive care beds without ventilator, three OTs, and one outpatient department (OPD). Additionally the orthopedic trauma facilities included a room for casting/splinting and one for physiotherapy.

Admission criteria were acute trauma, readmission of treated patients with complications, or a life-threatening condition of any cause.

### Implants

The implants used for orthopedic trauma surgery were intramedullary nails, external fixators, K-wires and plates. The Surgical Implant Generation Network (SIGN Fracture Care International, Richland, WA, USA) intramedullary nail was used. This nail can be used interchangeably for femur, tibia, and humerus. For proximal femoral fractures, a proximal femoral nail was used (Stryker Trauma AG, Selzbach, Switzerland). There were small and large external fixator systems (Hoffmann II external fixator system and Hoffmann II compact, Stryker Trauma AG, Selzbach, Switzerland, and AO external steel fixator, Depuy Synthes, Oberdorf, Switzerland), standard sized steel K-wires (1.2–3.5 mm) and Ender nails (4 mm), cerclage wires, and a set of small and large fragment low-contact steel plates (Braun Aesculap, Tuttlingen, Germany).

### Surgeries

The OT with C-arm was run routinely 5 days a week during daytime and for emergencies at night. In the other OTs, general surgical procedures or second and third look soft tissue surgeries were conducted.

### Epidemiology

Treatment and retrospective data acquisition were done for 3 months, from the 10th of October 2015 to the 8th of January 2016.

In this period, 282 patients were admitted, having 367 injuries. This results in 3.03 patients admitted per day due to trauma. 273 patients had 349 fractures.

On these 282 trauma patients, 263 orthopedic surgeries were performed in 64 days (4.11 osteosyntheses per day).

### Statistics

Continuous variables were expressed as means ± standard deviation, whereas categorical variables as percentages (%). The Kolmogorov–Smirnov test was used to assess distribution normality. For parametric variables, the Student’s *t* test was used for the comparison of two groups; for non-parametric variables the Mann–Whitney *U* test was implemented. Differences for categorical variables were assessed with the Chi-square test. Differences were considered statistically significant if the null hypothesis could be rejected with > 95% confidence (*p* < 0.05).

## Results

### Causes of trauma

The causes for trauma were road traffic accidents (RTA) for 215 patients (76.24%), falls for 59 (20.57%), falls from height for 6 (2.13%), and stab wounds for 3 (1.06%). RTAs include a large variety of mechanisms. Pedestrians got hit by moving vehicles at night because they walk on unlit roads. Some patients fell off a truck or children from motorcycles. Drivers or passengers mostly do not use seat belts and some crash into the front window or get ejected out of the car.

### Fractures in adults and children

Table 1 shows the amount of fractures, subdivided for fractures in adults and children, for open (Open) and closed (Closed) fractures, and for fractures treated with an osteosynthesis (OS), treated conservatively (Cons), and fractures being operated without osteosynthesis (Op).

**Table 1:** Listing of all fractures included in the study

Fracture	Total	Adults		Children		Adults			Children		
		Open	Closed	Open	Closed	OS	Cons	Op	OS	Cons	Op
Tibial shaft (AO 42)	97	52	34	8	3	63	13	10	9	1	1
Femoral shaft (AO 32)	53	5	31	0	17	30	4	2	4	13	0
Distal humerus (AO 13)	24	0	3	0	21	2	1	0	19	2	0
Forearm distal (AO 23)	17	2	7	0	8	4	4	1	8	0	0
Forearm shaft (AO 22)	15	3	8	1	3	9	2	0	2	1	1
Tibia proximal (AO 41)	15	5	8	0	2	7	4	2	0	2	0
Malleolar (AO 44)	14	5	9	0	0	9	3	2	0	0	0
Foot (AO 8)	14	7	4	3	0	7	1	3	2	0	1
Tibia distal (AO 43)	11	3	4	3	1	4	2	1	2	0	2
Maxillofacial	11	8	3	0	0	6	2	3	0	0	0
Femur distal (AO 33)	10	3	6	0	1	5	3	1	0	1	0
Hand (AO 7)	10	4	3	0	3	4	2	1	3	0	0
Pelvis (AO 61)	9	1	6	0	2	3	4	0	0	2	0
Humeral shaft (AO 12)	9	0	8	0	1	0	8	0	0	1	0
Femur proximal (AO 31)	8	0	8	0	0	2	6	0	0	0	0
Forearm proximal (AO 21)	7	4	3	0	0	6	1	0	0	0	0
Skull	5	3	1	1	0	0	1	3	0	0	1
Acetabulum (AO 62)	5	0	5	0	0	1	4	0	0	0	0
Patella (AO 34)	4	0	4	0	0	1	3	0	0	0	0
Spine (AO 5)	4	0	4	0	0	0	4	0	0	0	0
Humerus proximal (AO 11)	2	1	1	0	0	0	2	0	0	0	0
Clavicle (AO 15)	2	0	2	0	0	1	1	0	0	0	0
Complex elbow	2	1	0	0	1	0	0	1	1	0	0
Scapula (AO 14)	1	0	1	0	0	0	1	0	0	0	0
Total	349	107	163	16	63	164	76	30	50	23	6
Percentages	100.00	30.66	46.70	4.58	18.05	46.99	21.78	8.60	14.33	6.59	1.72

The most common fracture was the tibial shaft fracture (*n* = 97; 27.79%), followed by the femoral shaft fracture (*n* = 53; 15.19%), and the distal humerus fracture (*n* = 24; 6.88%).

### Open and closed fractures

Tibial shaft fractures were the most common open fractures (48.78%), followed by fractures of the foot (8.13%).

In adults, open tibial shaft fractures account for 48.60% of open fractures. Including the proximal tibial, the distal tibial, and the malleolar region, this amount rises to 60.75%; including the foot it rises to 67.29%.

In children, open tibial fractures account for 50.00% of open fractures, tibial fracture at any level for 68.75% and including the foot for 87.50%.

Femoral shaft fractures were the most common closed fractures (21.34%) followed by tibial shaft (16.37%) and supracondylar humerus fractures (10.62%).

In adults, 20.86% were closed fractures of the tibial shaft and 19.02% of the femoral shaft; in children, 33.33% were closed fractures of the distal humerus and 26.98% of the femoral shaft.

### Osteosynthesis

Table 2 shows the amount of closed and open fractures and the osteosynthesis techniques used. The most common osteosynthesis was the external fixator, followed by K-wires and the nail. In adults, the external fixator and the nail account for a similar high amount of osteosynthesis performed. In children, the K-wire fixation accounts for most osteosynthesis by far.

**Table 2:** Listings of fractures and percentages treated with different implants

	External fixator	Nail	K-wires	Plate	Screws	Total
All fractures (*n* = 349)						
Open fractures (*n* = 123)	59	9	13	5	2	88
Closed fractures (*n* = 226)	9	50	48	22	2	131
Total	**68**	**59**	**61**	**27**	**4**	219
Percentages	31.05	26.94	27.85	12.33	1.83	100.00
Fractures in adults (*n* = 270)						
Open fractures (*n* = 107)	50	9	12	5	2	78
Closed fractures (*n* = 163)	9	48	10	22	2	91
Total	**59**	**57**	**22**	**27**	**4**	169
Percentages	34.91	33.73	13.02	15.98	2.37	100.01
Fractures in children (*n* = 79)						
Open fractures (*n* = 16)	9	0	1	0	0	10
Closed fractures (*n* = 63)	0	2	38	0	0	40
Total	**9**	**2**	**39**	**0**	**0**	50
Percentages	18.00	4.00	78.00	0.00	0.00	100.00

The total numbers of fractures and used osteosynthesis technique are in bold

### Upper and lower extremity **(**hand and foot)

The fractured region was 89 times the upper extremity and 226 times the lower extremity. In the upper extremity, K-wires were used the most followed by plates and external fixators. In lower extremity, nails were used the most followed by external fixators, and K-wires. In hand and foot fractures (*n* = 24) mostly K-wires were used (*n* = 13; 54.17%) followed by conservative treatment (*n* = 8; 33.33%) and external fixators (*n* = 3; 12.50%). These fractures were open in 14 cases (58.33%). The external fixator was used for the lower extremity in 80.88%. In adults, this amount was 77.97% and in children 100.00%. For the tibial shaft, the external fixator was used in 67.79% in adults and 77.78% in children. K-wires were used for the upper extremity in 67.21%. In adults, this amount was 36.36% and in children 84.62%. In the lower extremity, excluding the foot (27.27%), their indication was mainly as a salvage procedure for fixation (36.36%). Plates were used in 51.85% for the upper extremity and in 44.44% for the lower extremity. The most common indication for plate osteosynthesis was forearm fractures (22.22%). In closed upper extremity fractures of adults, 73.33% of osteosyntheses were plates and the rest K-wires. Nails were used for the lower extremity only, femur and tibia. In adults, closed femur fractures were nailed in 93.55%, closed tibia fractures in 55.88%. In the closed lower extremity fracture, 67.61% of the osteosyntheses were nails (see Table 3).

**Table 3:** Percentages of implants used for adults and children, open and closed fractures

	All in %		Adults in %		Children in %	
	Open	Closed	Open	Closed	Open	Closed
External fixator	**67.05**	6.87	**64.10**	9.89	**90.00**	0.00
K-wires	14.77	36.64	15.38	10.99	10.00	**95.00**
Nail	10.23	**38.17**	11.54	**52.75**	0.00	5.00
Plate	5.68	16.79	6.41	24.18	0.00	0.00
Screws	2.27	1.53	2.56	2.20	0.00	0.00
	100.00	100.00	99.99	100.01	100.00	100.00

Percentages printed boldly show the most used implants

### Complications

There were 16 complications (5.67%) in all patients. There were five implant failures, one tibial malunion after conservative treatment, and one patient died during intubation after a stab wound in the face and neck. There were nine postoperative infections, seven in open fractures. They occurred in six cases after debridement and external fixator osteosynthesis (five tibial shaft, one malleolar, and one pelvic fracture) and once after debridement and fixation with a plaster of paris (one tibial shaft). The two infections in closed fractures occurred after plating a tibial head fracture and after K-wire osteosynthesis of a supracondylar fracture. This results in postoperative infection rate of 3.42% (2.66% for open and 0.76% for closed fractures).

## Discussion

Surgeons working in LIC need profound surgical skills focusing on injuries common in these countries [7]. A special interest of the surgeons should be on fractures of the tibial and femoral shaft in adults and the supracondylar humerus in children due to RTAs [15]. These fractures were the most common three fractures as they account for 49.86%. Open fractures below knee are the most common injuries. Basically, these fractures can be treated with three types of implants: external fixators, K-wires, and nails. The *external fixator* was used the most, mainly in open fractures. The use of it is common for an NGO setting in a LIC with civilian trauma or disaster surgery where open fractures occur frequently [2, 16]. The external fixator’s universal use makes it indispensable for this hospital. It also can be used for almost every fracture not amendable to other implants available. The local OT staff is familiar with its handling and the national surgeons are able to use it. The implant’s main disadvantage is its limited availability. K-wires were the major implants for children. In adults, they were used for the upper extremity and the foot. Using K-wires for intramedullary stabilization of diaphyseal long bones (elastic stable intramedullary nailing, ESIN), besides conventional techniques, makes them indispensable for fracture treatment in children. Nearly every closed fracture in children that needed osteosynthesis could be treated sufficiently. Its use for hand and foot fractures in adults increases its value. The *nail* is the first choice for closed tibia and femur fractures. Although we used a C-arm for nailing, its use can be omitted. Nailing was the only internal fixation procedure that was mastered by the local surgeons without international help. Moreover, the SIGN nail is free of charge and readily available. Nails should be used according the motto “nail what you can nail”. Plates were the least used implant. Their indications were fractures in adults of the forearm at any level, the proximal tibia, and the malleolar region. Plates were used mainly for fractures not treatable with any of the aforementioned implants, because the national surgeons were not able to use them without international help. The infection rate was higher in this Sierra Leonian hospital compared to high-income countries [10]. This comparison, however, is difficult because elective surgery was not performed in our hospital, the amount of open fractures is tenfold [5] compared to Europe, and the level of patients’ compliance is low. Although the OT is new, the OT staff needed to be guided for sterility. The high amount of incoming patients and the limited resources resulted in distinct overwhelming of the surgeon in charge, confusion, and sometimes in a complete loss of overview. Recommendations for the treatment of these patients could help the surgeon to cope with these problems. They should help national and international surgeons to make decisions for the management and treatment of patients.

### Proposal for decision-making (Fig. 1)

**Fig. 1:**
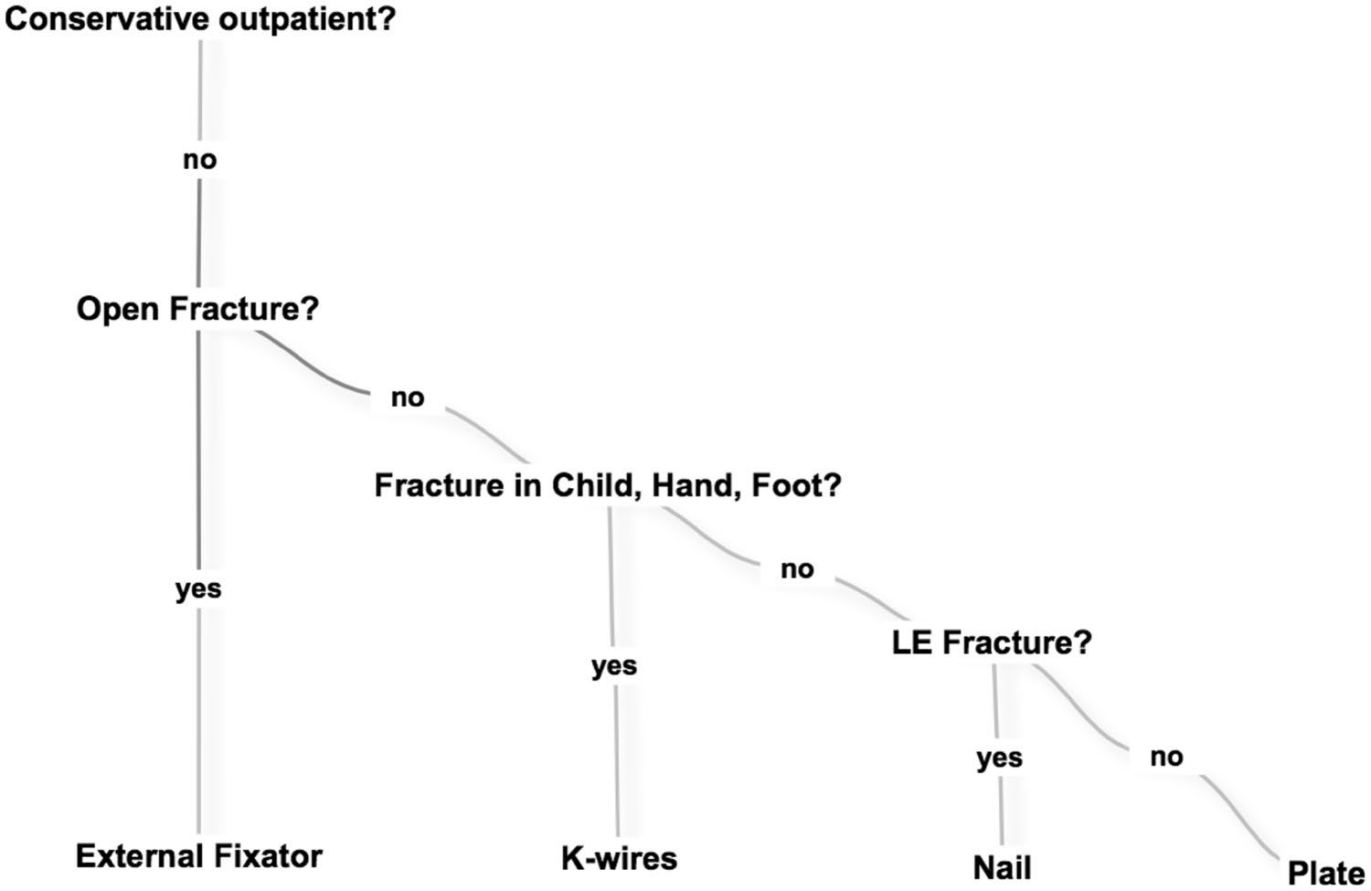
Algorithm for decision-making

A possible algorithm for trauma hospitals in a LIC should keep the amount of stationary patients low, cover the most common fractures, and preferably recommend implants easy to handle and available. A full or overloaded hospital can hardly provide sufficient operative care. This means that patient inflow needs to be reduced and outflow increased. This can be achieved by treating patients conservatively and as outpatients. A femoral shaft fracture in a child that is treated in traction will block a bed for at least 4 weeks compared to ESIN treatment where the patient can be discharged after 2 days. Admitted patients should be treated with an osteosynthesis to shorten the hospital stay. For surgery, only four implants need to be considered and three of them cover most of the fractures: external fixators, K-wires, and nails.

The fracture groups treated with these implants are, respectively, open fractures (35.24%), closed fractures in children or hand and foot (20.06%), and fractures of the lower extremity (29.80%). Fractures of the upper extremity (9.46%) can be treated with plates. In open fractures, 67.05% of osteosyntheses were external fixators. In closed fractures in children, hand and foot, 89.36% of osteosyntheses were K-wires. In closed fractures of the lower extremity in adults, 67.61% of osteosyntheses were nails. In closed fractures of the upper extremity in adults, 73.33% of osteosyntheses were plates. For the decision-making of fracture treatment the surgeon should answer the following consecutive questions (Fig. 1).

The algorithm represents a simple approach for a place where complex strategies should be minimized and decision made straightforward. The goal should not be to treat patients individually in the best possible way, but, as many patients as possible, sufficiently. To conserve resources, patients with fractures that can be treated on an outpatient basis should not be admitted and patients operated should be discharged as soon as possible. This is difficult in a LIC setting where compliance is low and the home of patients may lack of water, electricity and hygiene.

**Fig. 2:**
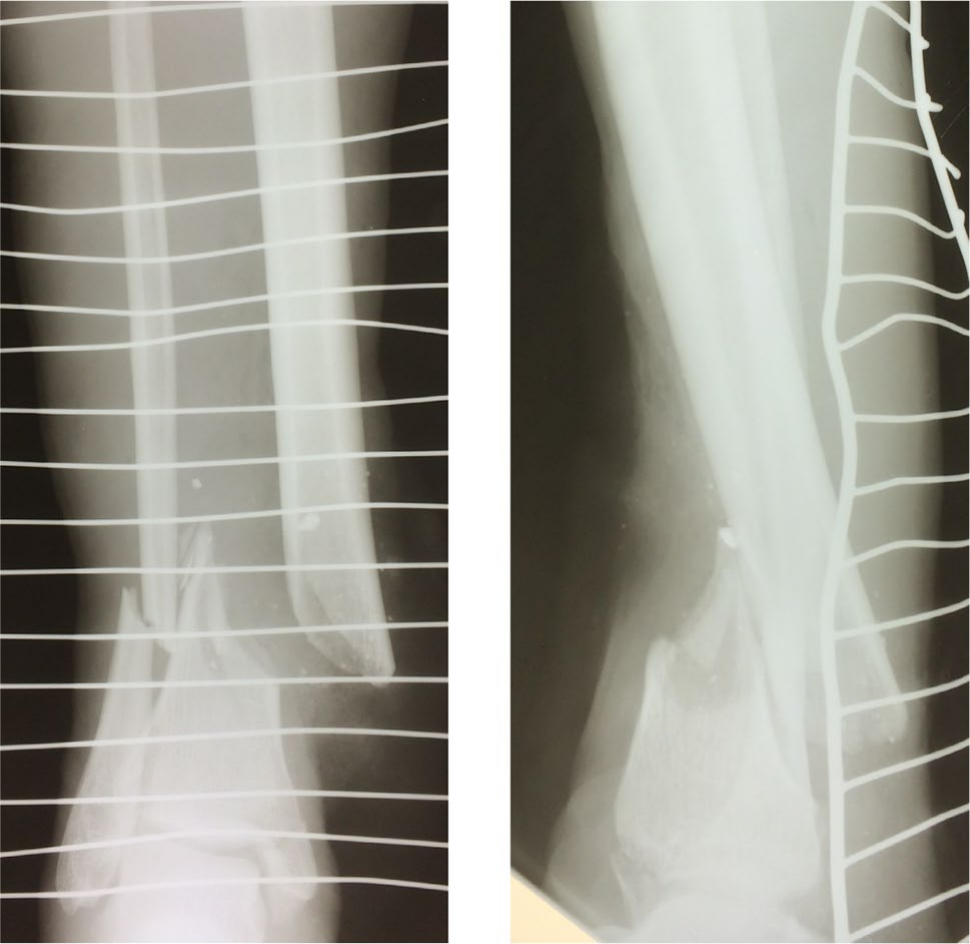
X-ray in two planes of a patient (45year-old male) with a right tibial shaft fracture 42 A2.3 III° B open

**Fig. 3:**
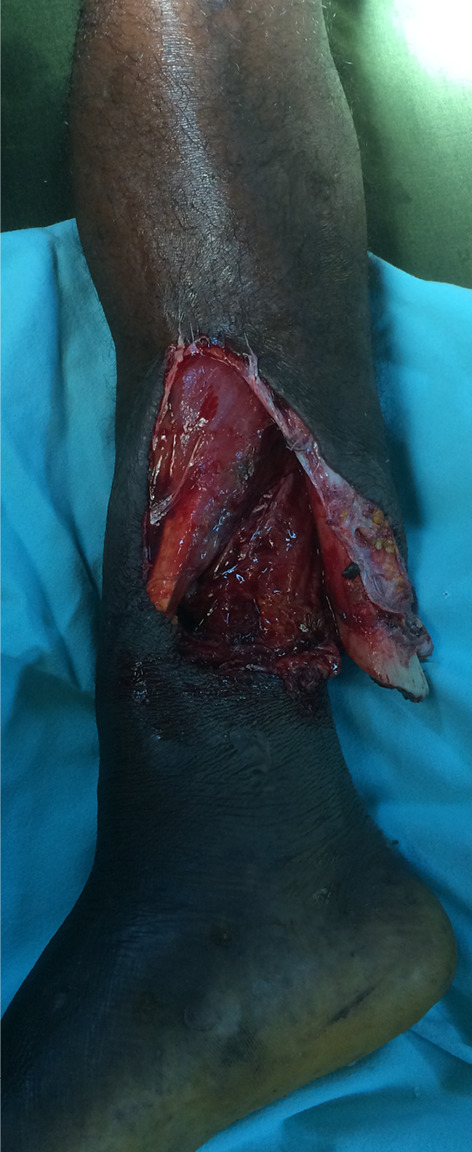
Clinical picture of the same patient as seen in Fig. 2

When deciding upon operative versus conservative treatment in Sierra Leone, the surgeon has to maintain flexibility and sometimes treat similar fractures differently. Instruments or implants might not be available or hospital beds may be occupied. The missing of implants or instruments impaired the surgeon’s ability to work, forcing him to adapt and to improvise. When external fixators ran out, fractures had to be fixed with K-wires (Figs. 2, 3) or with plaster of Paris instead. Some patients were waiting for an operation so long that the planned operation became useless. A clear cut for the indication operative versus conservative is impossible in these conditions and they might change from day to day.

Some fractured regions remain problems for the treating surgeon in this setting. These regions are mostly articular or metaphyseal fractures, as they mostly require plating.

Most of the studies investigating this topic focus on the evaluation of the high amount of trauma [8, 11] or on training national surgeons in LIC for orthopedic trauma [12, 21]. These studies all conclude and agree that trauma surgeons are desperately needed in these countries [14, 19]. Although studies for skills of general surgeons in mission were published [20], our work tries to present a practical approach for international and national surgeons focused on trauma.

## Conclusion

In a confusing austere environment where patients’ inflow overwhelms a single surgeon’s capacity, an algorithm could help to cope with the high amount of trauma. Questions that need to be answered for treatment are: Can the patient be treated outpatient conservatively? Is the fracture open? Is the fracture in a child? Is the fracture in the lower extremity?
